# Research on the Impact Mechanical Properties of Real-Time High-Temperature Granite and a Coupled Thermal–Mechanical Constitutive Model

**DOI:** 10.3390/ma16072773

**Published:** 2023-03-30

**Authors:** Yubai Li, Yue Zhai, Yifan Xie, Fandong Meng

**Affiliations:** 1 School of Geological Engineering and Geomatics, Chang’an University, Xi’an 710064, China; liyubai@chd.edu.cn (Y.L.);; 2 Key Laboratory of Mine Geological Hazard Mechanism and Control, Shaanxi Institute of Geological Survey, Xi’an 710054, China

**Keywords:** granite, thermal–mechanical coupling, SHPB, unified strength theory, constitutive model

## Abstract

Studying the mechanical behavior of rocks under real-time high-temperature conditions is of great significance for the development of energy caverns, nuclear waste disposal projects, and tunneling engineering. In this study, a real-time high-temperature impact compression test was conducted on Sejila Mountain granite to explore the effects of temperature and external load on its mechanical properties. Based on the concepts of damage mechanics and statistics, a coupled thermal–mechanical (T-M) damage constitutive model was established, which considers the temperature effect and uses the double-shear unified strength as the yield criterion. The parameter expressions were clarified, and the accuracy and applicability of the model were verified by experimental data. The research results indicated that high temperatures had an obvious damaging and deteriorating effect on the strength of the granite, while an increase in impact velocity had an enhancing effect on the strength of the granite. The established constitutive model theoretical curve and test curve showed a high degree of agreement, indicating that the coupled T-M model can objectively represent the evolution process of damage in rocks and the physical meaning of its parameters is clear.

## 1. Introduction

The development and utilization of geothermal resources, the deep burial disposal of nuclear waste, and tunneling engineering involve real-time high-temperature rocks. Compared with room-temperature conditions, the stress status of rocks in a high-temperature environment usually changes, which affects the stability of a rock mass [1,2]. In addition, underground rock engineering often involves explosion-proof structural engineering, excavation and blasting, etc., and it is necessary to predict and control the damage degree of rocks under impact load. Therefore, it is of great theoretical significance and practical value to study the law governing the impact mechanical properties of rocks under real-time high temperatures for underground rock engineering.

The physical and mechanical properties of rocks are greatly affected by temperature and impact load [3,4]. With the increase in the strain rate, the energy absorption rate of a rock increases, the damage degree increases, and the peak stress is affected differently by the rock particle size [5,6,7]. Kumari et al. studied the effect of temperature on the mechanical behavior of rock specimens through triaxial tests under high temperature and high pressure and found that the type of rock instability is affected by the temperature and mainly characterized by a brittle–plastic transformation of the rock [8]. Because the rock will acquire brittleness after being subjected to a high temperature, the mechanical properties of the rock after cooling, following a high-temperature treatment, are significantly different from those during the high-temperature treatment. At present, real-time high-temperature impact mechanical testing is mainly carried out by three test methods. The first method consists in heating the specimen and part of the pressure bar simultaneously [9]. Although the change of the wave impedance of steel elastic pressure bars caused by high temperature is corrected during data processing, the experimental error caused by the effects of the high temperature on the yield strength and elastic modulus of the bar is still unavoidable. The second method consists in placing temperature-insensitive spacers at both ends of the specimen [10]. Only the spacer block is heated during the test. This method can effectively improve the drawbacks of the first method but it has higher requirements for the material of the spacer block, the test equipment is more complicated, and the operation is cumbersome. The third test method consists in heating only the specimens in a furnace [11]. The specimens reaching the prefabrication temperature are transferred to the split Hopkinson Pressure Bar (SHPB) device, and the dynamic mechanical test is completed. This method must be completed in a short time to avoid heat transfer after the bar comes in contact with the specimen. If the heat loss is too large during the transfer process, temperature compensation should be considered. Due to the limitation of the test technology, the results of real-time high-temperature impact mechanical tests have large errors, and the analysis and understanding of the impact mechanism of rock damage are controversial [12,13,14].

Various scholars have conducted a deep study on the failure mechanism of thermally damaged rocks by applying new mechanical theories, in order to define an appropriate method to describe the stress–strain relationship in response to rock damage [15]. Since Dougill [16] introduced the concept of damage mechanics into the field of rock mechanics, damage mechanics’ principles have become important for studying rock mass damage. For example, Cao et al. [17] divided the rock into two parts, damaged and undamaged, and proposed a damage constitutive equation considering rock hardening and softening. Mubarak et al. [18] analyzed the damage constitutive model of a rock under cyclic loading conditions. Deng et al. [19] proposed a damage constitutive model based on continuous damage mechanics and the maximum entropy distribution of micro-element strength. However, the above models cannot accurately describe the mechanical behavior of rocks at high temperature. Therefore, it is necessary to build a constitutive model of rocks subjected to high temperature considering the effects of temperature. Liu and Xu [20] carried out a study on the change of the main mechanical parameters of granite (20~600 °C), obtained the fitting equation of uniaxial compressive strength and the Poisson’s ratio of granite depending on the temperature, and used them to define thermal damage. Rong et al. [21] defined thermal damage from the perspective of acoustic emission and established a damage coupling equation considering the crack closure effect. Zhang et al. [22] were the first to propose a thermal–mechanical (T-M) factor and established a one-dimensional nonlinear coupling model. At present, constitutive models considering the temperature effects are mainly divided into three types. The first type is the Thermo–Elasto–Plastic damage model based on the damage theory and Thermo-Elasticity theory [23,24]. The second type is a rheological model based on creep tests and the Thermo-Viscoplastic theory [25,26]. The third type is the thermal damage constitutive model of rocks based on statistical distribution [27,28]. The statistical damage constitutive model can directly and accurately describe the evolution process of rock damage, so as to better describe the mechanism of rock damage. Therefore, this model has obvious advantages over the other two models. Due to the randomness in the evolution of the internal defect distribution in a rock, it is generally considered that it satisfies the Weibull distribution [29] or the normal distribution [30], and the damage discriminating variable is usually expressed by the strength yield criterion. The strength theories include the maximum normal stress strength theory, the maximum shear stress theory, the maximum normal strain theory, the octahedral shear stress theory [31], the MC strength theory [32], the DP strength theory [33], the Griffith and modified Griffith theory [34], the HB empirical strength theory [35], etc. In addition, Xie et al. [36] used the method of continuous damage mechanics to study the thermal damage of rocks when the temperature changed and established a rock thermal damage failure criterion based on the energy theory. Yu and He [37] put forward the double-shear unified strength theory of materials on the basis of the double-shear stress idea, which has strong applicability and has been applied in some practical projects [38,39,40].

To sum up, the existing studies mostly focused on the mechanical properties of rocks under the action of a single field, and research on the mechanical properties of rocks under T-M coupling is less developed. How to reduce the experimental error and make the experimental results more convincing, and how to rationally analyze the experimental results through theory are still in the exploratory stage. Therefore, in this study, Sejila Mountain granite was used as the test material, and an impact compression test under real-time high temperature was carried out through the improved SHPB system. The changes in granite mechanical properties at different temperatures and impact velocities are discussed, and a coupled T-M constitutive model of granite based on the double-shear unified strength yield criterion is established to provide theoretical support for high-temperature rock engineering-related calculations and numerical simulations.

## 2. Materials and Methods

### 2.1. Specimen Preparation

**Figure 1:**
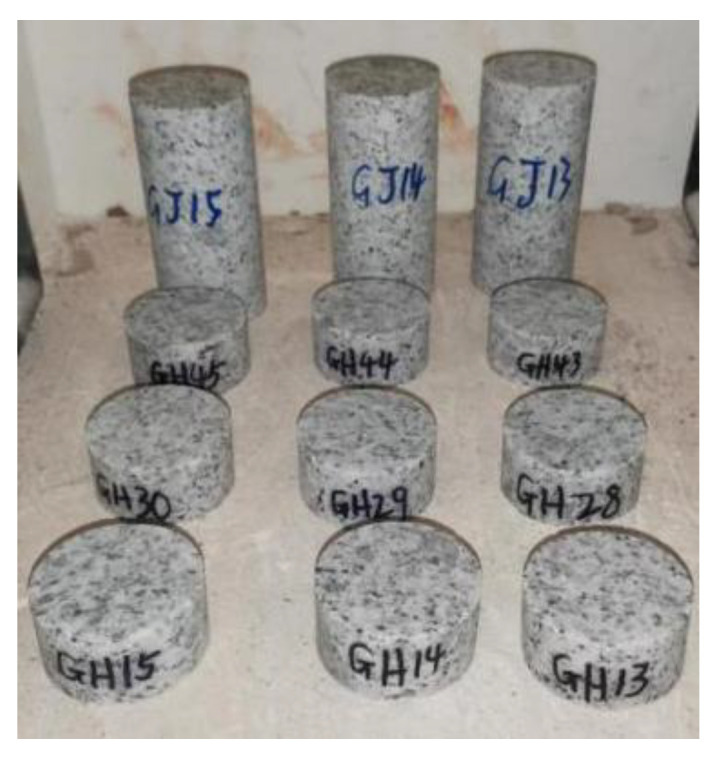
Granite specimens.

The granite specimens were collected from the tunnel engineering site near Sejila Mountain, China. The surface was gray-white with black particles, the particles were dense, and the specimens were well cemented and free of cracks. According to International Society for Rock Mechanics (ISRM), an impact compression test specimen is processed into a cylinder of Φ48 mm × 25 mm, and a static compression test specimen is processed to obtain a sample of Φ48 mm × 100 mm. A double-sided grinding machine was used for fine processing to ensure that the parallelism error was less than 0.05 mm, and the error of the height and diameter of the specimen was not more than 0.3 mm. The maximum deviation of the end face perpendicular to the axis of the specimen did not exceed 0.25° (Figure 1) [41].

### 2.2. Test Equipment and Test Methods

**Figure 2:**
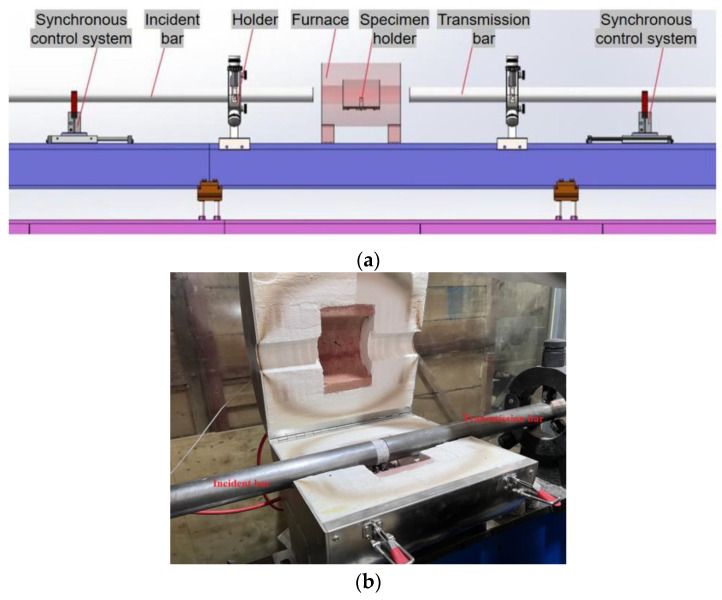
SHPB test system with an intelligent real-time high-temperature furnace. (**a**) Abridged general view, (**b**) real-time high-temperature furnace.

The test adopted the SHPB system with a pressure bar diameter of 50 mm and an intelligent real-time high-temperature furnace. The schematic diagram is shown in Figure 2. The elastic modulus of the pressure bars was 210 GPa, the density was 7850 kg/m^3^, the propagation velocity of the stress wave was 5170 m/s, the length of the impact bar was 400 mm, the length of the incident bar was 4400 mm, and the length of the transmission bar was 3000 mm. In this experiment, five working conditions were chosen, i.e., room temperature, 200 °C, 400 °C, 600 °C, and 800 °C. The heating rate was 10 °C/min during the heating process. After reaching the target temperature, the temperature was kept constant for 2 h to ensure that specimens were heated evenly. After the specimens were heated, the control system pushed the incident bar and the transmission bar to both ends of the specimen synchronously, and the impact test was completed in the real-time high-temperature furnace. This method effectively avoids heating the pressure bar and thus affecting its mechanical parameters and also avoids the temperature loss caused by the transfer of the specimen. The control pressure in this test was 0.2 MPa, 0.4 MPa, and 0.6 MPa, and the corresponding initial impact velocities were 5.4 m/s, 8.8 m/s, and 11.3 m/s. Three sets of specimens were prepared for all working conditions, and the mechanical tests were repeated three times. The median of the test data was selected for the analysis.

Based on the one-dimensional stress wave assumption and the homogeneity assumption, the three-wave method [42,43] was used to calculate the raw data:

(1) $$\{\begin{array}{c} \dot{\varepsilon }=-\frac{\mu_{1}-\mu_{2}}{l_{s}}=\frac{C_{0}}{l_{s}}\left(-\varepsilon_{i}+\varepsilon_{r}+\varepsilon_{t}\right)\\ \varepsilon =\frac{C_{0}}{l_{s}}\left(-\varepsilon_{i}+\varepsilon_{r}+\varepsilon_{t}\right)d\tau \\ \sigma =\frac{EA}{2A_{s}}\left(\varepsilon_{i}+\varepsilon_{r}+\varepsilon_{t}\right) \end{array}$$

where $\dot{\varepsilon }$ is the strain rate, $\varepsilon_{i}$ is the incident wave strain, $\varepsilon_{r}$ is the reflected wave strain, $\varepsilon_{t}$ is the transmitted wave strain, $C_{0}$ is the one-dimensional elastic wave velocity in the pressure bar, $l_{s}$ is the length of the impact bar, E is the elastic modulus, $A_{s}$ and A are the cross-sectional area of the specimen and the pressure bar, respectively.

### 2.3. Results

**Figure 3:**
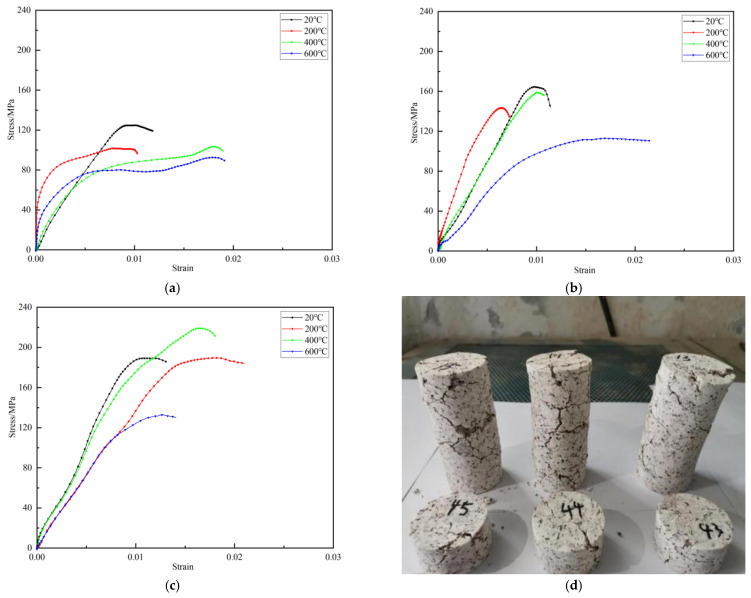
Stress–strain curves at different temperatures with specimens treated at 800 °C. (**a**) Stress–strain curves at different temperatures (5.4 m/s), (**b**) stress–strain curves at different temperatures (8.8 m/s), (**c**) stress–strain curves at different temperatures (11.3 m/s), (**d**) granite specimens treated at 800 °C.

The stress–strain curves of the granite specimens at different temperatures and impact velocities were determined, as shown in Figure 3. At 800 °C, the granite specimen had lost its bearing capacity. Since this occurrence did not meet the assumptions of the SHPB test, the impact mechanical test of the specimen under this working condition was not carried out.

It can be seen in Figure 3 that the test curves have no obvious compaction section, and the slope of some curves rises sharply at the initial stage. Although molybdenum-based grease was applied to both sides of the specimen before the test, the data oscillation phenomenon caused by the uneven contact surface would still occur at the moment when the incident bar hit the specimen, which directly led to a sharp increase in the deformation modulus. For such phenomena, it should be considered that the dynamic deformation modulus between the zero point and 50% of the peak stress was always less than or equal to the dynamic deformation modulus at 50% of the peak stress point in the analysis of the test data. Differently from what observed at 8.8 m/s and 11.3 m/s, the test curve at 5.4 m/s impact velocity showed an obvious approximate plateau before reaching the peak stress, which is because when the impact velocity is low, the shaping effect on the wave is far less than that of a high-velocity impact. The incident wave cannot be well approximated to a sine wave, but the compressive strength value and strain rate of the corresponding specimen will be improved. At the same time, it was also shown that even if the external load reached the maximum impact resistance of the specimen, due to the extremely short action time, there was a short adaptation stagnation period after the rock was broken, resulting in resistance to the peak load for a short time. In addition, this also showed that the granite underwent a certain degree of brittle–plastic transformation at high temperature. At the same impact velocity, the peak strain at high temperature increased compared with that at room temperature, which once again indicated that the brittleness of the rock began to change to plasticity under the action of high temperature. Under the same impact velocity, the peak stress at 200 °C was lower than that at room temperature, while the peak stress at 400 °C was significantly higher than that at 200 °C, the peak stress at 600 °C was significantly reduced, and the bearing capacity was lost at 800 °C. Before 400 °C, due to the complete evaporation of the water absorbed in the minerals inside the specimen and the release of water from the crystal lattice, the effective stress increased. The strength of granite increased with the increase of the effective stress. However, the loss of water from the specimen also reduced its strength to some extent. Therefore, before reaching 400 °C, the peak strength of the specimen fluctuated due to the interaction of multiple mechanisms [44]. At 600 °C, quartz undergoes a phase transition, and the volume of the crystal structure suddenly increases, resulting in a large, unbalanced force around the cracks, which manifests as a rapid decline in the strength of the specimen macroscopically [45]. A temperature higher than 600 °C commonly leads to mineral intragranular displacement and intergranular dislocation [46], and the rapid development of fractures leads to a sharp drop in the peak stress of granite and to the rapid deterioration of its mechanical properties.

At the same temperature, compared with 5.4 m/s, the peak stress of the specimens increased significantly when the impact velocity was 8.8 m/s and 11.3 m/s and as the impact velocity increased. The influence of the initial damage caused by the high temperature on the curve was significantly reduced, and the impact velocity replaced the influence of high temperature on the curve, becoming dominant. It can be seen that the dynamic impact mechanical properties of granite were the result of the coupling between high temperature and impact velocity, and as the impact velocity increased, the impact of the initial damage caused by the high temperature on mechanical properties such as peak stress decreased.

## 3. Constitutive Model

### 3.1. Thermal Damage Variable

When studying mechanical properties such as the deformation, strength, and damage of rocks at high temperature, in addition to exploring their regularity from an experimental point of view, it is important to establish a coupled T-M damage model from a theoretical point of view. Similar to other rocks, granite is generally considered to be an aggregate of minerals. When the temperature increases, thermal stress makes the mineral particles inside granite expand and squeeze, causing fracture expansion and connection, resulting in thermal damage. A large number of experiments have proved that a high temperature has a softening effect on granite, which is shown by the decrease of the elastic modulus with the increase in temperature [47,48,49]. Therefore, the elastic modulus is a function of temperature and s can be chosen to reflect the thermal damage variable $\emptyset_{T}$, which is defined as

(2) $$\emptyset_{T}=1-E_{T}/E_{0}$$

where $E_{T}$ is the elastic modulus at temperature T; $E_{0}$ is the initial elastic modulus of the rock.

### 3.2. Mechanical Damage Variable

Granite has initial defects during its natural formation, and when it is loaded, microfractures in its internal structure continue to develop. The change of the microstructure leads to the formation of macroscopic cracks, which continue to expand, and finally macroscopic damage occurs. Therefore, the formation and development of microcracks are the root cause of the damage. A large number of studies proved that the evolution of the defect distribution in a rock satisfies a statistical law, and the strength of micro-elements approximately obeys the Weibull distribution [50]; the distribution density function $h_{\left(w\right)}$ is:

(3) $$h_{\left(w\right)}=\frac{m}{w_{0}}{\left(\frac{w}{w_{0}}\right)}^{m-1}exp\left[-{\left(\frac{w}{w_{0}}\right)}^{m}\right]$$

where w is the discriminant variable of micro-element damage; m and $w_{0}$ are the parameters in the Weibull distribution.

The mechanical damage variable $\emptyset_{M}$ can be written as:

(4) $$\emptyset_{M}=\int_{0}^{w}h_{\left(w\right)}dw=1-exp\left[-{\left(\frac{w}{w_{0}}\right)}^{m}\right]$$

### 3.3. Coupled T-M Damage Constitutive Model

According to the above analysis, the total T-M damage ∅ is:

(5) $$\emptyset =\emptyset_{T}+\emptyset_{M}-\emptyset_{T}\emptyset_{M}$$

Based on Lemaitre’s principle and Hooke’s law, the constitutive relation is:

(6) $$\sigma =E_{0}\varepsilon \left(1-\emptyset \right)=E_{0}\varepsilon \left(1-\emptyset_{T}\right)\left(1-\emptyset_{M}\right)=E_{T}\varepsilon exp\left[-{\left(\frac{w}{w_{0}}\right)}^{m}\right]$$

where ε is the axial strain, σ is the axial stress of the rock.

In this study, a strength yield criterion was considered to represent w. Yu and He [37] proposed a unified strength theory based on the double-shear yield criterion. Starting from a unified physical model, the theory takes into account all stress components and their different effects on material failure and can be applied to various geotechnical materials. Both the M-C strength theory and the double-shear strength theory are special cases and contain new calculation criteria that are more reasonable than the D-P criterion [33]. The unified strength theory based on the double-shear stress yield criterion is expressed as:

(7) $$w=\{\begin{array}{c} \frac{1-a}{3a}I_{1}+\frac{1-b}{1+b}\sqrt{J_{2}}sin\theta +\frac{2+a}{a\sqrt{3}}\sqrt{J_{2}}cos\theta ,\;0^{{}^{\circ}}\leq \theta \leq \theta_{b}\\ \frac{1-a}{3a}I_{1}+\frac{a+ab+b}{a\left(1+b\right)}\sqrt{J_{2}}sin\theta +\frac{2+a+ab-b}{\sqrt{3}\left(a+ab\right)}\sqrt{J_{2}}cos\theta ,\;\theta_{b}\leq \theta \leq 60^{{}^{\circ}} \end{array}$$

where $I_{1}$ is the first invariant of the stress tensor, $J_{2}$ is the second invariant of the stress tensor, θ is the shear stress angle, *a* and *b* are the parameters of the corresponding function. Among them, $I_{1}$ and $J_{2}$ can be expressed as:

(8) $$\{\begin{array}{c} I_{1}=\sigma_{1}^{\prime }+\sigma_{2}^{\prime }+\sigma_{3}^{\prime }\\ J_{2}=\frac{1}{6}\left[{\left(\sigma_{1}^{\prime }-\sigma_{2}^{\prime }\right)}^{2}+{\left(\sigma_{2}^{\prime }-\sigma_{3}^{\prime }\right)}^{2}+{\left(\sigma_{3}^{\prime }-\sigma_{1}^{\prime }\right)}^{2}\right] \end{array}$$

and

(9) $$\begin{array}{c} \sigma_{1}^{\prime }=\frac{\sigma_{1}E\varepsilon_{1}}{\sigma_{1}-\mu \sigma_{2}-\mu \sigma_{3}}\\ \sigma_{2}^{\prime }=\frac{\sigma_{2}E\varepsilon_{1}}{\sigma_{1}-\mu \sigma_{2}-\mu \sigma_{3}}\\ \sigma_{3}^{\prime }=\frac{\sigma_{3}E\varepsilon_{1}}{\sigma_{1}-\mu \sigma_{2}-\mu \sigma_{3}} \end{array}$$

*a* and *b* can be expressed as:

(10) $$\{\begin{array}{c} a=\frac{1-sin\theta }{1+sin\theta }\\ b=\frac{\left(1+a\right)\tau_{0}-\sigma_{t}}{\sigma_{t}-\tau_{0}} \end{array}$$

In the above equation, $\tau_{0}$ is the shear strength, $\sigma_{t}$ is the tensile strength, μ is the Poisson’s ratio, *E* is the elastic modulus.

By combining Equations (7)–(10), the unified strength theory expressed in the form of principal stress can be obtained. Under uniaxial compression test conditions ($\sigma_{2}^{\prime }$ = $\sigma_{3}^{\prime }$ = 0), the unified strength theory based on the double-shear stress yield criterion can be expressed as:

(11) $$w=\frac{E\varepsilon_{1}\left(\sigma_{1}/a-\sigma_{3}\right)}{\sigma_{1}-2\mu \sigma_{3}}=\frac{E\varepsilon_{1}}{a}$$

Substituting Equation (11) into Equation (6), the damage evolution model of the specimen can be obtained, which is expressed as:

(12) $$\sigma =E_{T}\varepsilon exp[-{\left(\frac{\left(1-sin\theta \right)E_{0}\varepsilon }{\left(1+sin\theta \right)w_{0}}\right)}^{m}]$$

### 3.4. Constitutive Model Parameters Determination

The mechanical parameters of the high-temperature rock statistical damage model proposed in this study include $E_{T}$, $E_{0}$, and φ and can be measured by routine rock tests. Therefore, choosing method to determine the distribution parameters $w_{0}$ and *m* was the key problem to solve the application of the statistical damage constitutive model. At present, the methods for determining distribution parameters are mainly the inversion analysis method [51], the linear fitting method [52], and the peak point method [53]. The peak point method focuses on the highest point of the test curve due to its clear physical meaning and simple processing. The influence of other test points on a rock stress–strain curve can be weakened, and the problem that the statistical damage constitutive equation has no mathematical meaning within a certain range can be avoided. The stress–strain curve obtained by theoretical calculation completely depends on the properties of the statistical damage constitutive equation itself. It requires the model to satisfy the following governing equations:

(13) $$\{\begin{array}{c} {\sigma_{\left(\varepsilon \right)}|}_{\varepsilon =\varepsilon_{c}}=\sigma_{c}\\ {\frac{\delta \sigma }{\delta \varepsilon }|}_{\sigma =\sigma_{c},\varepsilon =\varepsilon_{c}}=0 \end{array}$$

where $\sigma_{c}$ and $\varepsilon_{c}$ are the peak stress and peak strain of the stress–strain curve, respectively.

Combining Equations (6) and (13):

(14) $$\{\begin{array}{c} m=1/\left[ln\left(E_{T}\varepsilon_{c}\right)-ln\sigma_{c}\right]\\ w_{0}=\frac{E_{0}\varepsilon_{c}}{a}m^{1/m} \end{array}$$

It can be seen from Equation (14) that the model parameters *m* and $w_{0}$ can be obtained from the experimental parameters $E_{0}$, $\sigma_{c}$ and $\varepsilon_{c}$, which shows that the mechanical parameters of the high-temperature rock statistical damage constitutive model proposed in this study are easy to obtain, and their physical meaning is clear.

## 4. Model Validation

**Figure 4:**
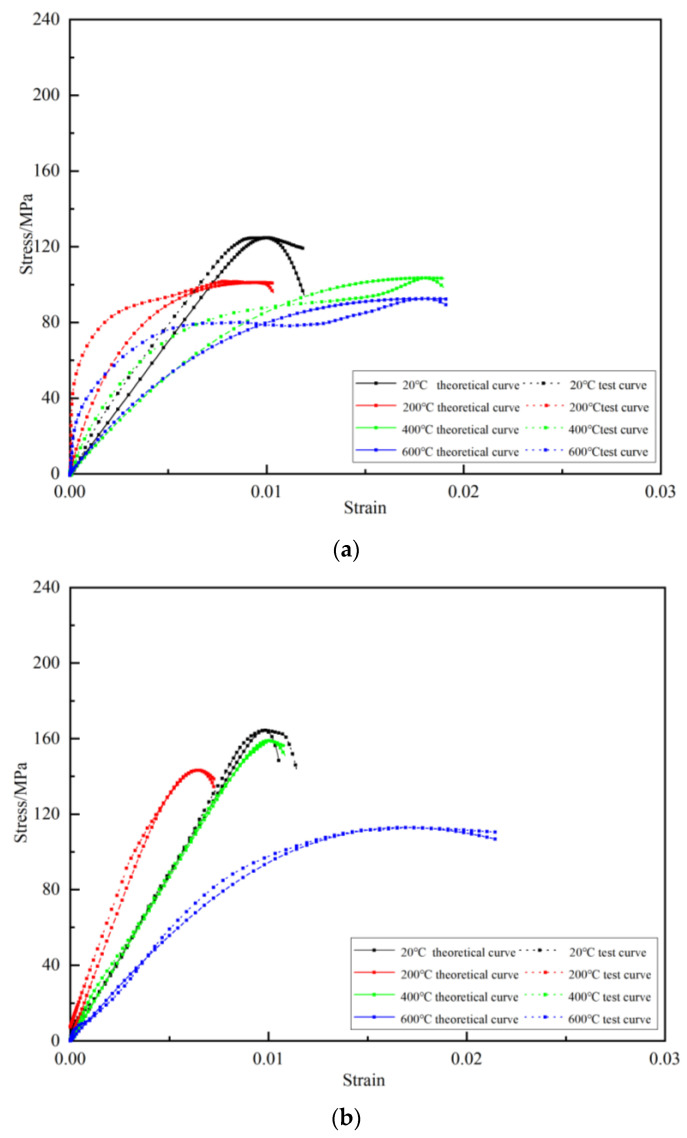

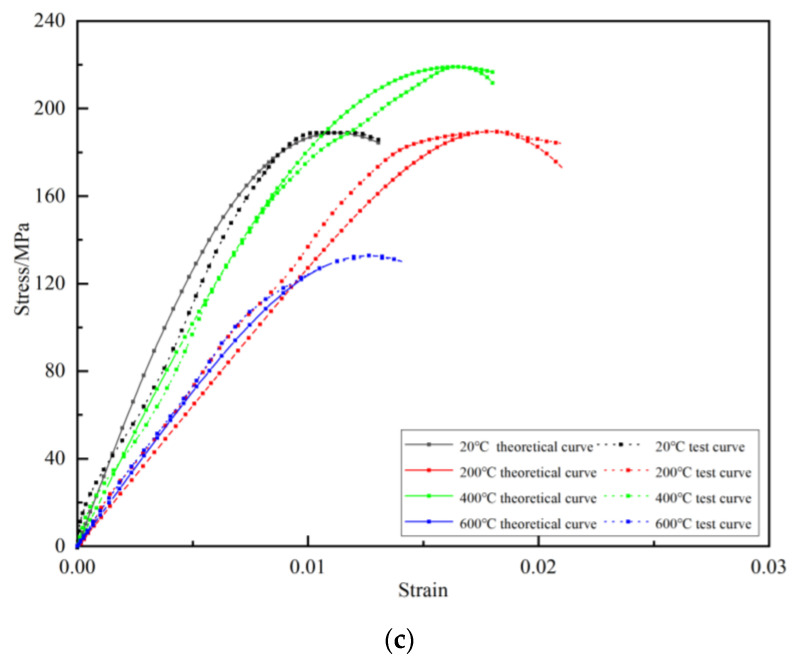
Test curve and theoretical curve fitting. (**a**) Stress–strain curves at different temperatures (5.4 m/s), (**b**) stress–strain curves at different temperatures (8.8 m/s), (**c**) stress–strain curves at different temperatures (11.3 m/s).

In order to analyze the rationality and applicability of the above-described statistical damage constitutive model, the test results were fitted with the model curve. In this study, the RMT-150 C rock mechanical test system was used to conduct static compression tests at room temperature and obtain the initial mechanical properties of the granite specimen. The initial elastic modulus was 42.873 GPa, the Poisson’s ratio was 0.160, and the peak stress was 186.351 MPa. At room temperature, the longitudinal wave velocity of the Sejila Mountain granite was 2770 m/s, and the rock internal friction angle was 45°. According to the above test results and the parameter determination method, the parameters of the model built are shown in Table 1. Among them, the dynamic elastic modulus was calculated by taking two points corresponding to 40% and 60% of the peak stress. The theoretical curve of the rock stress–strain relationship at different temperatures could be obtained from Equation (12), as shown in Figure 4.

As shown in Figure 4, the theoretical curve obtained according to the statistical damage constitutive model showed the same general trend as the experimental curve, which reflects the changes of granite strength with temperature. It shows that the coupled T-M damage constitutive model based on the unified strength theory proposed in this study can reflect the deformation characteristics of granite under different temperatures and uniaxial compression tests. It can also well characterize the stress–strain curve under different temperatures. However, there is also a certain deviation, which is within a reasonable range. The reason is that the viscous characteristics of the rock in the impact test were not considered in the process of establishing the equation and would improve the dynamic strength of the rock [54]. The curve was generally lower than the experimental curve.

Compared with the results at 5.4 m/s, we found that the theoretical curve fitting effect was better when the impact velocity was 8.8 m/s and 11.3 m/s. This is because the wave shaper improved the smoothness of the stress wave at the expense of partial strain rate and compressive strength. At 5.4 m/s, in order to more truly reflect the mechanical properties of the specimen itself, the selected wave shaper could not shape well the rectangular wave generated by the impact into an approximate sine wave, resulting in a long plateau segment in the test curve.

**Figure 5:**
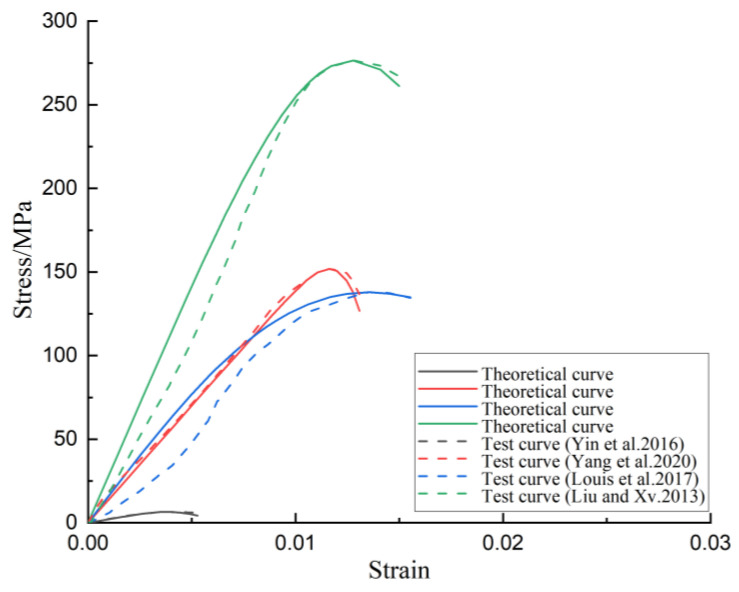
Comparison of experimental results in literature and theoretical curves [55,56,57,58].

In order to further illustrate the rationality and universality of the theoretical model based on the double-shear unified strength theory, the uniaxial impact compression test results of marble and coal under real-time high temperature from Yin et al., Yang et al., Louis et al., and Liu and Xv. were compared with ours [55,56,57,58]. The results obtained after the model was fitted are shown in Figure 5. It can be seen that the theoretical results obtained in this research showed the same general trend as the experimental results of other scholars. They could better reflect the variations of the rock pre-peak intensity at different temperatures, and the error was within a reasonable range. The error was because in the process of compressive deformation of the rock, there was sometimes a compaction section before entering the elastic section. At this stage, the pores of the rock itself were compacted, causing the initial shape of the curve to sag, which is inconsistent with the theoretical curve.

## 5. Discussion

M coupling is a process in which two physical fields, the stress field and the temperature field, influence each other, that is, the temperature has an influence on the stress deformation, and the stress deformation also affects the temperature. In other words, T-M coupling requires the coexistence of a stress field and a temperature field and refers to the study of a rock mechanical properties under real-time high temperature, rather than to the study of a rock mechanical properties after a high-temperature treatment. There is an essential difference between the two. Studies have shown that the brittleness of granite is restored after applying a high temperature, and the mechanical properties of a rock specimen change suddenly only when the brittle–plastic transition occurs in specimen. Therefore, the deterioration of the mechanical properties of rock specimens under the action of real-time high temperature proceeds continuously, while the mechanical behavior of rock specimens after cooling following a high-temperature treatment shows a sudden change, which is closely related to the phase transition in the rock structure.

It can be seen in this study that the coupling effect was nonlinear and changed continuously throughout the loading process. The effect of temperature on granite damage was obvious. At the same impact velocity, the mechanical damage increased with the increase of the temperature. This is because the thermal expansion coefficients of the minerals contained in granite are different, and the physical properties such as the thermal elasticity of minerals behave differently in different directions, that is, anisotropy. This can lead to an uncoordinated thermal expansion across particle boundaries. Another reason is that as the temperature rises, the thermal motion of the molecules increases, resulting in the weakening of the force between molecules, causing the lattice to dislocate or crack.

**Figure 6:**
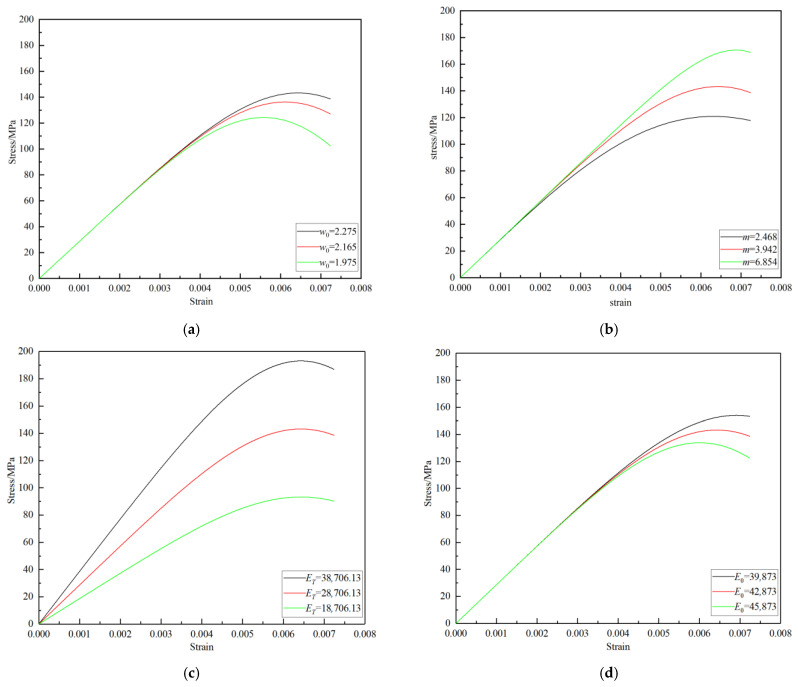
Influence of the model parameters on the stress–strain curves. (**a**) Effect of the parameter $w_{0}$ on the theoretical curve, (**b**) effect of the parameter m on the theoretical curve, (**c**) effect of the parameter $E_{T}$ on the theoretical curve (**d**) effect of the parameter $E_{0}$ on the theoretical curve.

According to the constitutive model established above, the determination of $w_{0}$, *m*, $E_{T}$, and $E_{0}$ was the key issue in building the model. In order to clarify the influence of each parameter on the stress–strain curve, the influence of the model parameters on the theoretical curve is here further discussed by taking the specimen at 200 °C under 8.8 m/s as an example and analyzing the results shown in Figure 6.

It can be seen in Figure 6 that with the decrease of the model distribution parameters $w_{0}$ or *m*, the peak stress and peak strain of the specimen both decreased significantly, but there was no difference in the initial deformation stage of the specimen. Therefore, the model parameters $w_{0}$ and *m* mainly characterize the strength characteristics of the specimen without affecting its lithological characteristics such as the elastic modulus. The parameter $E_{T}$ mainly affected the slope and peak stress of the loading section of the stress–strain curve. The increase of $E_{T}$ correspondingly increased the slope and peak stress of the loading section of the stress–strain curve, but the peak strain was not affected. The effect of changes in the parameter $E_{0}$ on the theoretical curve was exactly opposite to that of changes in distribution parameter. With the decrease of $E_{0}$, the peak stress and peak strain of the stress–strain curve both increased.

For this constitutive model, this study used the extreme value method to determine the model parameters according to the rock yield criterion. This determination method is suitable for the solution of loading tests at different temperatures, allows the easy determination of a rock mechanical parameters, and is convenient for engineering applications. In the process of establishing the rock statistical thermal damage model, the mechanical parameters and statistical distribution parameters used in the model have nothing to do with lithology, so this model is also applicable to other rocks with different lithologies.

It is worth noting that the statistical T-M coupling constitutive model of rocks proposed in this study ignores the influence of primary defects of the rock and the partial closure of micro-cracks after heating. Therefore, the initial compaction segment of the stress–strain curve cannot be represented. In addition, the value of each parameter of this model has nothing to do with the post-peak point, and there is no constraint on the post-peak point; therefore, the model cannot describe the mechanical behavior of a specimen after failure.

## 6. Conclusions

In this study, a real-time high-temperature impact compression test was conducted on Sejila Mountain granite using the SHPB system and an intelligent synchronous high-temperature furnace. The variation laws of peak stress, peak strain, and elastic modulus were revealed, and a coupled T-M damage constitutive model based on the unified strength theory was established. The main conclusions are as follows: 

The ultimate temperature bearing capacity of Sejila Mountain granite was found to be less than 800 °C. When the temperature exceeded 400 °C, the stress decreased significantly with the increase of the temperature. The peak strain at high temperature was higher than that at room temperature. At the same temperature, an increase in impact velocity had a significant enhancing effect on the strength of the specimens. 

The coupled T-M damage constitutive model established based on the unified strength theory can fully reflect the strength characteristics of a rock before failure, can well describe the stress–strain curves at different temperatures, and provides curves with a high degree of fitting with the experimental curves. 

The model parameter $E_{T}$ has a significant effect on the peak stress and slope of the theoretical curve, but not on the peak strain. The distribution parameters of the model mainly characterize the strength characteristics of a specimen. With a decrease in the distribution parameters, the peak strength and peak strain of the theoretical curve decrease significantly. The effect of the parameter $E_{0}$ on the theoretical curve is exactly the opposite of that of the distribution parameter.

## Figures and Tables

**Table 1:** Values of the model parameters.

	Temperature/°C	$E_{T}$/MPa	$w_{0}$	m	$\sigma_{c}$	$\varepsilon_{c}$
5.4 m/s	20	13,979.2	3.161	9.215	124.66	0.00994
	200	45,206.58	1.393	0.693	101.09	0.00947
	400	11,665.95	5.743	1.141	103.43	0.018
	600	13,504.7	4.651	1.036	92.52	0.01799
8.8 m/s	20	17,581.72	2.818	21.762	164.40	0.00979
	200	28,706.13	2.275	3.942	143.23	0.00643
	400	17,289.46	3.087	11.700	158.89	0.01001
	600	11,940.04	5.794	1.719	112.92	0.01692
11.3 m/s	20	28,106.52	3.952	1.948	188.91	0.01123
	200	12,836.02	6.139	5.196	189.44	0.01789
	400	21,098.09	5.850	2.198	218.99	0.01636
	600	14,346.63	4.542	3.210	132.80	0.01264

## Data Availability

The data that support the findings of this study are available on request from the corresponding author.
